# Ibrexafungerp, a Novel Oral Triterpenoid Antifungal in Development: Overview of Antifungal Activity Against *Candida glabrata*

**DOI:** 10.3389/fcimb.2021.642358

**Published:** 2021-03-11

**Authors:** Ahmed Gamal, Sherman Chu, Thomas S. McCormick, Katyna Borroto-Esoda, David Angulo, Mahmoud A. Ghannoum

**Affiliations:** ^1^ Department of Dermatology, Case Western Reserve University, Cleveland, OH, United States; ^2^ College of Osteopathic Medicine of the Pacific, Northwest (COMP), Lebanon, OR, United States; ^3^ Scynexis Inc., Jersey City, NJ, United States; ^4^ Department of Dermatology, Center for Medical Mycology, University Hospitals Cleveland Medical Center, Cleveland, OH, United States

**Keywords:** ibrexafungerp, *Candida glabrata*, ß-(1,3)-D-glucan, triterpenoid class (fungerps), antifungal

## Abstract

Systemic infections caused by *Candida* species are an important cause of morbidity and mortality among immunocompromised and non-immunocompromised patients. In particular, *Candida glabrata* is an emerging species within the *Candida* family that causes infections ranging from superficial to life-threatening systemic disease. Echinocandins and azoles are typically the first-line therapies used to treat infections caused by *C. glabrata*, however, there is an increasing prevalence of resistance to these antifungal agents in patients. Thus, a need exists for novel therapies that demonstrate high efficacy against *C. glabrata*. Ibrexafungerp is a first-in-class glucan synthase inhibitor with oral availability developed to address this increasing antifungal resistance. Ibrexafungerp demonstrates broad *in vitro* activity against wild-type, azole-resistant, and echinocandin-resistant *C. glabrata* species. Furthermore, ibrexafungerp has shown efficacy in low pH environments, which suggests its potential effectiveness in treating vulvovaginal candidiasis. Additional preclinical and clinical studies are needed to further examine the mechanism(s) of ibrexafungerp, including acting as a promising new agent for treating *C. glabrata* infections.

## Introduction

The incidence of fungal infections has increased significantly over the last several decades and are important causes of morbidity and mortality (Lass-Florl, 2009). *Candida* species are the most common cause of invasive fungal infections and are associated with high mortality and morbidity rates among both immunocompromised and non-immunocompromised hosts (Dodds Ashley et al., 2012; Pfaller M. et al., 2012). Although *Candida albicans* is the most prevalent *Candida* species isolated from humans, *Candida glabrata* infections have increased significantly over the last two decades and are now one of the primary causes of candidemia in the United States, accounting for more than a third of all candidemia isolates (Bassetti et al., 2006; Chakrabarti et al., 2009; Lockhart et al., 2012; Matsumoto et al., 2014). The reason for the increasing trend of *C. glabrata* infections is multifactorial and can be due to geographical variation, differing patient populations, and increased use of prophylactic fluconazole (Richardson and Lass-Florl, 2008).

In addition to systemic infections, superficial infections caused by *Candida* are also important.

In particular, vulvovaginal candidiasis (VVC) is the second most common cause of vaginitis after bacterial vaginosis (Anderson et al., 2004). The incidence of VVC is substantial, with estimations of up to 70–75% of women experiencing an episode of VVC and it has been estimated that approximately 10–15% of asymptomatic women are colonized with a *Candida* species (Sobel, 2007). *C. glabrata* is the second most common species identified in women with VVC after *C. albicans* with a prevalence ranging between 3.4 to 20% in America, Europe, and Australia (Corsello et al., 2003; Holland et al., 2003; Sojakova et al., 2004; Richter et al., 2005; Grigoriou et al., 2006; Paulitsch et al., 2006). Several studies have reported higher prevalence of *C. glabrata* causing VVC (29.5–50.4%), specifically in studies performed in Asian and African countries (Okungbowa et al., 2003; Cetin et al., 2007; Mohanty et al., 2007; Ahmad and Khan, 2009). Additionally, older women, women with uncontrolled diabetes, and women afflicted with HIV have been associated with high percentages of *C. glabrata* species causing VVC (Goswami et al., 2000; Corsello et al., 2003; Dan et al., 2006; Fan et al., 2008). Published studies suggested that *C. glabrata* infections emerge as breakthrough infections in women following a maintenance low-dose fluconazole prophylaxis. It is believed that recurrent vulvovaginal candidiasis has a mutual relationship with non-*albicans Candida* infections, where the occurrence of one predisposes to others. Moreover, recurrent infections result in increased frequency of non-*albicans Candida* colonization, while the persistence of these non-*albicans Candida* can also lead to recurrent infections (Fidel et al., 1999; Makanjuola et al., 2018).

## Resistance

Despite *C. glabrata* lacking several virulence factors associated with other *Candida* species, such as hyphal growth and the ability to secrete proteases, this species is a growing concern for mucosal and systemic bloodstream infections (Kaur et al., 2005; Lim et al., 2012; Sardi et al., 2013). An active population-based surveillance for culture-confirmed candidemia was conducted by the Centers for Disease Control and Prevention (CDC) during 2012–2016 in four U.S. states (Georgia, Maryland, Oregon, and Tennessee). Out of 3,492 cases of candidemia, *C. glabrata* accounted for the second highest organism detected, resulting in 28% of cases compared to *C. albicans* that accounted for 38% (Toda et al., 2019). A major concern is that *C. glabrata* resistance to currently available antifungals is increasing and the emergence of multidrug resistance threatens the effectiveness of both azoles and echinocandins (Pfaller and Diekema, 2007). In general, resistance to antifungal agents may be either intrinsic, such as the resistance of *C. krusei* to fluconazole, or acquired—as a result of extensive use of antifungal agents (Arendrup and Patterson, 2017).

*C. glabrata* has been found to have higher rates of azole antifungal resistance in comparison to other *Candida* species, such as *C. albicans, C. tropicalis, and C. parapsilosis* (Lockhart et al., 2012). In a study published in 2012, two hundred ninety-one (291) *C. glabrata* isolates from around the world demonstrated low susceptibility when tested against a panel of antifungal agents including triazoles (Castanheira et al., 2014). This may be due to the widespread use of azoles, especially in patients using these agents for long term prophylaxis and treatment. This, in turn, results in the emergence of intrinsically less susceptible strains of *C. glabrata* (Redding et al., 2004). Various mechanisms of resistance to azoles have been described in the literature including gene mutation (*ERG11* mutation causing less binding), gain of function mutation (GOF), gene up-regulation (GOF in *UPC2* leading to overexpression of *ERG11* causing insufficient azole activity), and overexpression of efflux pumps (decreasing the intracellular concentrations of the drug) (Sanglard, 2016). In this regard, *CgCDR1* and *CgCDR2* transporters are found to be the main mechanism for azole resistance in *C. glabrata* which occur as a result of CgPDR1 GOF mutation (Ghannoum and Rice, 1999; Borst et al., 2005; Vale-Silva et al., 2013; Sanglard and Coste, 2016).

Additionally, *C. glabrata* resistance to echinocandins is increasingly prevalent with rates ranging between 3 and 30% (Alexander et al., 2013). This finding is particularly noted in patients who are extensively using echinocandins, especially in strains exhibiting less susceptibility to fluconazole and other azoles (Beyda et al., 2014). Resistance to echinocandins has only been linked to mutations in two hot spot regions of the *FKS* genes leading to amino acid substitutions in these regions (hot spots 1 and 2 of *FKS1* gene for *C. albicans* and hot spot 1 and 2 of *FKS1* and *FKS2* gene for *C. glabrata*) (Sanglard, 2016). Echinocandins susceptibility test was evaluated against a total of 1,380 isolates of *C. glabrata* collected between 2008 and 2013 from four U.S. cities. Screening for *FKS* mutations was done in 1,032 isolates including 77 isolates showing intermediate or resistant MIC values to at least one echinocandin. A total of 51 isolates were found to have *FKS* mutations, 15 isolates with *FKS1* mutations and 35 with *FKS2* mutations. It was also found that not all *FKS* mutations cause the same degree of resistance which is demonstrated by the variable MICs among these isolates (Pham et al., 2014). Within *FKS1*, mutations at S629 caused higher MICs values than others occurring at R631 or D632. Furthermore, mutations at R631 were associated with higher micafungin MICs but lower values for caspofungin and anidulafungin. Additionally, D632V mutations demonstrated elevated MIC values to all echinocandins, which was not the case for the D632Y mutation where high MIC values were not reported for any of the echinocandins tested.

A major concern is the potential development of multidrug resistant (MDR) *C. glabrata* strains. In a study based on the SENTRY Antimicrobial Surveillance Program for the years 2006 through 2010 and the Centers for Disease Control and Prevention population-based surveillance conducted in 2008–2010, out of 1,669 *C. glabrata* isolates, 162 were resistant to fluconazole including 18 isolates that were also resistant to one or more of the echinocandins. In all 18 isolates, mutations in either *FKS1* or *FKS2* were detected (Pfaller M. A. et al., 2012). Furthermore, in a population-based active surveillance for culture-confirmed candidemia in four sites in the U.S. during 2012–2016, out of 929 C*. glabrata* isolates, 12 isolates were MDR (Toda et al., 2019).

In addition to resistance mechanisms described previously, a newly proposed mechanism that may explain the rapid acquisition of fluconazole, echinocandin, and amphotericin B resistance is *MSH2* DNA mismatch repair gene mutations. Out of 357 geographically diverse *C. glabrata* strains, these mutations were detected in nearly half of the isolates (susceptible and resistant). Interestingly, these mutations were more frequent among fluconazole-resistant and MDR *C. glabrata* isolates (Healey et al., 2016).

The increasing resistance to azoles and echinocandins in addition to amphotericin B toxicity and lack of oral bioavailability of these drugs calls for the development of new antifungals. Additional compelling support for the development of new effective antifungal to combat infection caused by *C. glabrata* is the recent report of amphotericin B resistant *C. glabrata* strains (Farmakiotis et al., 2014; Cho et al., 2015).

## Ibrexafungerp

### Mechanism of Action

Ibrexafungerp (formerly SCY-078) is the first compound of the enfumafungin-derived triterpenoid class of antifungals and the first oral (1-3)-β-D-glucan synthase inhibitor (GSIs) (Ghannoum et al., 2020a). GSIs are a group of drugs that act by inhibition of β-1,3-D-glucan synthase, a key enzyme in the biosynthesis of ß-(1,3)-D-glucan, a major component of the fungal cell wall.

β-1, 3-D-glucan synthase is unique to lower eukaryotes thus, antifungal agents that share this fungal-specific mode of action tend to exhibit low toxicity. GSIs were first introduced in 2001, with caspofungin the first echinocandin to be approved. Two additional echinocandins were later introduced, micafungin and anidulafungin (Heasley et al., 2012). The main advantages of echinocandins are broad-spectrum and fungicidal activity with a safety profile better than other antifungal agents. However, the key limitation of the echinocandins is the requirement for administration by intravenous infusion hence there is a need for new agents that can be administered orally with the preservation of these features (Walker et al., 2011).

Natural compounds screening led to the discovery of enfumafungin, a triterpene glycoside natural product, which was found to have *in vitro* antifungal activity comparable to caspofungin. However, it showed limited stability *in vivo*. Derivatives of enfumafungin were found to be potent inhibitors of β-1,3-D-glucan synthase and are structurally different from the echinocandins. Semi-synthetic modification of these derivatives resulted in improvement of oral bioavailability and PK properties thereby leading to the discovery of ibrexafungerp (Ghannoum et al., 2020a).

The aim of ibrexafungerp development is to be the first oral and IV GSI that can be used in the treatment and prevention of various fungal infections including life-threatening infections (Davis et al., 2020) as well as superficial ones (e.g., vulvovaginal candidiasis). Ibrexafungerp and echinocandins act by inhibition of β-1, 3-D-glucan synthase; however, they are different in structure and interact differently with the target enzyme resulting in a lower rate of resistance to ibrexafungerp (Jimenez-Ortigosa et al., 2017). Furthermore, the binding site for both appears to be nonidentical. In a recent study, ibrexafungerp demonstrated high activity against *Candida* species including wild-type and echinocandin-resistant strains in the presence of *FKS* mutations suggesting limited potential for cross-resistance with echinocandins (Pfaller et al., 2017). However, the identification of the binding site is currently under investigation.

### *In Vitro* and *In Vivo* Activity

Ibrexafungerp displayed potent *in vitro* activity against a wide range of *Aspergillus* species and *Candida* isolates, including isolates resistant to azoles and echinocandins (Jimenez-Ortigosa et al., 2014; Ghannoum et al., 2018). It also showed consistent activity against *C. glabrata* with *fks* mutations, which confer resistance to echinocandins, with MIC values ranging from <0.03 to 4 μg/ml (Nunnally et al., 2019). Additionally, ibrexafungerp has uniform and potent activity against *C. auris*, an emerging multidrug-resistant fungus, which was demonstrated in several studies (Berkow et al., 2017; Arendrup et al., 2020; Barat SB-E, 2020; Ghannoum et al., 2020a). Furthermore, ibrexafungerp has also shown the ability to reduce *C. auris* tissue fungal burden in an *in vivo* guinea pig model (Ghannoum et al., 2020b).

Interestingly, studies showed that ibrexafungerp inhibited cell growth and division of *C. auris*. These data suggest that in addition to the glucan synthase inhibition effect, this drug may have a separate target or may affect this enzyme through different mechanisms (Larkin et al., 2017). Ibrexafungerp has fungicidal activity against *Candida* species which was demonstrated in a time-kill study in which ibrexafungerp showed activity comparable to that of caspofungin. It also showed potent *in vitro* activity against *Candida* species in a low pH environment suggesting a therapeutic advantage of ibrexafungerp in the treatment of vaginal *Candida* infections (Larkin et al., 2019).

### Pharmacokinetics

Several studies were done to determine pharmacokinetic parameters and bioavailability of ibrexafungerp. *In vitro* bioavailability was assessed using Caco-2 cell monolayers as a predictor of absorption across the gut. The mean apparent permeability was 8.9 ± 0.78 × 10^−6^ cm/s for 5 μM ibrexafungerp indicating a good oral absorption. This was consistent with the data obtained following oral administration in animal models which displayed an oral bioavailability of 35% - 50% (calculated by oral AUC_0–12_ versus intravenous AUC_0–∞_) (Wring et al., 2017). It also demonstrated high protein binding (99.5–99.8%) in different species, which is expected for a compound with a lipophilic nature.

Ibrexafungerp achieved maximum plasma concentrations (C_max_) between 4 and 6 h reflecting a prolonged absorption phase after oral administration. In animals, observation of drug levels after a single dose showed a monophasic decline with a half-life of 7–9 h (Lepak et al., 2015; Wring et al., 2019a). A Phase 1 study in healthy volunteers demonstrated peak plasma concentration within 4–6 h with a linear decline and a mean terminal half-life of approximately 20–30 h. In the same setting, administration of ibrexafungerp with a high-fat meal showed increased bioavailability, on the other hand, food delayed the absorption of ibrexafungerp from a median time to peak concentration (Tmax) of 4.0 h in the fasted state to 6.0 h in the fed state (Trucksis et al., 2009).

In animals, ibrexafungerp displayed wide tissue distribution with a steady-state volume of distribution (Vdss) of >5 liter/kg (Wring et al., 2019a), which is several-fold greater than fluconazole and echinocandins (Lepak et al., 2015). Furthermore, assessment of the distribution of ibrexafungerp between plasma and kidney tissue displayed a tissue concentration 20- to 25-fold greater than that seen in plasma, indicating high tissue penetration. In comparison with micafungin, the mainstay in the treatment of intra-abdominal candidiasis, ibrexafungerp demonstrated superior lesion penetration properties in mice with intra-abdominal candidiasis (Lee et al., 2020). After a single oral dose of ibrexafungerp (15 mg/kg) in rats, ratios of tissue-to-blood AUC in commonly affected organs by invasive fungal disease were: spleen 54-fold; liver 50-fold; lung 31-fold; bone marrow 25-fold; kidney 20-fold; skin 12-18-fold; vaginal tissue 9-fold; and skeletal muscle 4-fold, with minimal distribution to central nervous system tissues (Wring et al., 2019a). Ibrexafungerp showed a high potential to accumulate in vaginal tissue and fluids with a tissue concentration 2-5 folds higher than plasma concentration. Carbon labeled [^14^C] ibrexafungerp was used to determine the elimination route for ibrexafungerp in animal models following intravenous (I.V.) and oral administration.

Most of the radioactivity (~80%) was recovered from feces for both routes while nearly 1% was recovered from urine. Total recovery of the radioactivity of the administered I.V. and oral doses was 92.2 and 91.8% respectively (Larkin et al., 2019).

Safety of ibrexafungerp was evaluated in a total of 12 subjects that received a loading dose of 1250-mg ibrexafungerp on day 1 followed by daily maintenance doses of 750-mg SCY 780 citrate on days 2 and 3. Blood samples were collected predose and postdose on both days 1 and 3. Adverse events (AE) were reported in 6 subjects that were mostly in the form of diarrhea, abdominal pain, nausea, vomiting, and flatulence. However, these AE were mild or moderate in intensity with no serious AE reported and no subjects discontinued the study due to an AE (Wring et al., 2019b).

There was no clinically relevant effect on the QTc interval in healthy volunteers at a plasma concentration up to 4 μg/ml (Murphy et al., 2017). Ibrexafungerp also did not affect fertility or early embryonic development of male and female rats using doses above clinically efficacious doses which suggest potential use in the treatment of fungal infection in women during pregnancy (Carruthers et al., 2018).

*In vitro* studies demonstrated that ibrexafungerp is a CYP3A4 substrate and reversible inhibitor of CYP2C8 and CYP3A4. However, coadministration of a single-dose of ibrexafungerp with rosiglitazone (CYP2C8 substrate), and tacrolimus (CYP3A4 substrate) showed minimal effect on the pharmacokinetics of both drugs, suggesting low potential for ibrexafungerp to cause CYP-mediated drug interactions at therapeutic exposures. Moreover, data showed that co-administration of a single dose of tacrolimus with ibrexafungerp at a steady-state has no effect on the maximum blood level of tacrolimus with only a 1.4-fold increase in systemic exposure to tacrolimus which is markedly weaker interaction than observed with azoles. These findings are supportive for coadministration of ibrexafungerp and tacrolimus without the need for initial dose adjustment for tacrolimus (Wring et al., 2018; Wring et al., 2019b).

### Clinical Development Overview

Four clinical trials, CANDLE-304, SCYNERGIA, CARES, and FURI, are ongoing for further evaluation of ibrexafungerp. CANDLE-304 is a phase 3 study to evaluate the efficacy and safety of oral ibrexafungerp in patients with recurrent vulvovaginal candidiasis (VVC) ([[NoAuthor]]), SCYNERGIA is a multicenter randomized double-blind study to evaluate the safety and efficacy of coadministration of ibrexafungerp with voriconazole in patients with invasive pulmonary aspergillosis (A Multicenter, Randomized, Double-Blind Study to Evaluate the Safety and Efficacy of the Coadministration of SCY-078 With Voriconazole in Patients With Invasive Pulmonary Aspergillosis [Internet]), CARES is an open-label study to evaluate oral ibrexafungerp as an emergency use treatment for patients with candidiasis including *C. auris* candidemia (Open-Label Study to Evaluate the Efficacy, Safety, Tolerability and Pharmacokinetics of Oral Ibrexafungerp (SCY-078) as an Emergency Use Treatment for Patients With Candidiasis, Including Candidemia, Caused by Candida Auris [Internet]) and FURI is an open-label study to evaluate the efficacy and safety of ibrexafungerp in patients with fungal diseases refractory to standard treatment (Open-Label Study to Evaluate the Efficacy and Safety of SCY-078 (Ibrexafungerp) in Patients With Fungal Diseases That Are Refractory to or Intolerant of Standard Antifungal Treatment (FURI) [Internet]).

## Ibrexafungerp and *Candida glabrata*

### *In Vitro* Activity

Ibrexafungerp has been tested *in vitro* against *C. glabrata* isolates with *FKS1* and *FKS2* mutations, which have varying degrees of susceptibility to echinocandins. The *in vitro* activity of ibrexafungerp was determined for 34 C*. glabrata* clinical isolates with genotypic, phenotypic, or clinical echinocandin resistance (Schell et al., 2017). The MIC_90_ (minimum concentration that inhibited 90% of tested isolates) for ibrexafungerp against resistant *C. glabrata* was 4 μg/ml, while fluconazole and caspofungin showed MIC_90_ values of 128 and 8 μg/ml, respectively. Anidulafungin and micafungin exhibited MIC_90_ values of 2 μg/ml for both drugs against *C. glabrata*, while voriconazole showed MIC_90_ values of 4 μg/ml. In 23 C*. glabrata* clinical isolates from episodes of bloodstream infections, MIC_90_ for ibrexafungerp was 1 μg/ml with fluconazole demonstrating MIC_90_ values of 64 μg/ml (Schell et al., 2017).

In 89 echinocandin resistant *C. glabrata* isolates, MIC values of ibrexafungerp ranged from <0.03 μg/ml to 4 μg/ml with MIC_50_ (minimum concentration that inhibited 50% of tested isolates) and MIC_90_ values of 0.25 μg/ml and 1 μg/ml, respectively (Nunnally et al., 2019). MIC values for echinocandins among the same isolates ranged from 0.03 to 4 μg/ml for anidulafungin, 0.03 to >16 μg/ml for caspofungin, and 0.008 to >16 μg/ml for micafungin. Another study assessing 87 C*. glabrata* isolates showed mean MIC distributions ranging from 0.31 to 8 and 0.03 to 2.52 μg/ml for ibrexafungerp and micafungin, respectively (Jimenez-Ortigosa et al., 2017).

In 39 wild-type *C. glabrata* isolates, MIC values of ibrexafungerp ranged from 0.25 to 1 μg/ml with a modal MIC of 0.5 μg/ml (Pfaller et al., 2017). In the same isolates, modal MIC values of anidulafungin, caspofungin, and micafungin were 0.06, 0.06, and 0.015 μg/ml, respectively. In 28 echinocandin resistant isolates, ibrexafungerp MIC values ranged from 0.12 to 16 μg/ml with a modal MIC value of 1 μg/ml. In comparison, anidulafungin, caspofungin, and micafungin showed MIC ranges of 0.015 to 4, 0.03 to 16, and ≤0.008 to 4 μg/ml, respectively, with modal MIC values of 1, 0.5, and 0.06 μg/ml, respectively.

In two independent studies that evaluated over 400 clinical *Candida* species isolates collected between 2005 and 2015, *in vitro* MIC data was gathered to determine the activity of ibrexafungerp against multi-drug resistant *C. glabrata* strains with resistance to both echinocandins and fluconazole. A total of 17 C*. glabrata* isolates were resistant to echinocandins and fluconazole. MIC values for ibrexafungerp, caspofungin, micafungin, and fluconazole were ≥0.5, ≥2, ≥0.25, and ≥64 μg/ml, respectively. Ibrexafungerp was found to be active *in vitro* against 12/17 (71%) of *C. glabrata* resistant strains tested (Borroto-Esoda et al., 2017a).

A collection of 72 C*. glabrata* strains from hospitals throughout the United Kingdom between January 2015 and March 2016 were utilized to assess the *in vitro* activity of ibrexafungerp. Against echinocandin resistant *C. glabrata* isolates, MIC_50_ and MIC_90_ values were both 1 μg/ml for ibrexafungerp and amphotericin B. Anidulafungin and caspofungin had MIC_50_ and MIC_90_ values of 0.25 and 1 μg/ml, respectively. The MIC_50_ and MIC_90_ of micafungin were 0.06 and 0.25 μg/ml, respectively. For fluconazole, the MIC_50_ and MIC_90_ values were 2 and 32 μg/ml, respectively. Additionally, voriconazole and posaconazole MIC_50_ values were 0.125 and 0.5 μg/ml, respectively, while they both had a MIC_90_ value of 1 μg/ml (**Table 1**) (Borroto-Esoda et al., 2017b).

**Table 1:** MIC values (μg/ml) of ibrexafungerp and comparators against 72 echinocandin-resistant *C. glabrata* isolates (Lass-Florl, 2009).

Antifungal	IBX	AMB	ANID	CASP	MICA	FLZ	VORI	POSA
MIC_50_	1	1	0.25	0.25	0.06	2	0.125	0.5
MIC_90_	1	1	1	1	0.25	32	1	1

A compilation across five independent studies was performed to identify the *in vitro* MIC data for ibrexafungerp against *C. glabrata* isolates with *fks* mutations (30 *fks*1 and 49 *fks*2). Ibrexafungerp showed activity against 78% of *C. glabrata* isolates with *fks* mutations. Additionally, ibrexafungerp demonstrated greater *in vitro* activity compared to micafungin and caspofungin against *fks* mutated *C. glabrata* strains. The MIC_50_ of ibrexafungerp against *fks* mutant *C. glabrata* ranged from 0.5 to 1 μg/ml, MIC_50_ of caspofungin ranged from 0.5 to 4 μg/ml, and micafungin ranged from 0.125 to 0.25 μg/ml (Barat et al., 2018).

### *In Vitro* Activity at Different pH Levels

Larkin et al. demonstrated the efficacy of ibrexafungerp in lower pH environments by evaluating the activity of the drug against *Candida* species isolated from patients with vulvovaginitis (Larkin et al., 2019). The *in vitro* activity of ibrexafungerp was shown to be enhanced in lower pH environments. Against ten *C. glabrata* isolates, the MIC_50_ values of ibrexafungerp tested at pH levels of 7.0, 5.72, and 4.5 were 1, 0.5, and 0.063 μg/ml, respectively. In comparison, the MIC_50_ value of micafungin remained the same regardless of pH (0.25 μg/ml), while the MIC_50_ values of fluconazole at pH levels of 7.0, 5.72, and 4.5 were 1, 8, and 8 μg/ml, respectively (**Table 2**).

**Table 2:** *In vitro* activity of ibrexafungerp and comparators in lower pH environments (Dodds Ashley et al., 2012).

Antifungal	Ibrexafungerp			Micafungin			Fluconazole		
PH Level	**7.0**	**5.72**	**4.5**	**7.0**	**5.72**	**4.5**	**7.0**	**5.72**	**4.5**
MIC_50_ (μg/ml)	1	0.5	0.063	0.25	0.25	0.25	1	8	8

### Effect Against Biofilms

In a study performed in Madrid, Spain, the antifungal activity of ibrexafungerp, micafungin, and fluconazole was evaluated against planktonic and sessile *Candida albicans* and non-*albicans*; including 31 C*. glabrata* (Marcos-Zambrano et al., 2017). Antifungal susceptibility testing was performed using the Clinical and Laboratory Standards Institute (CLSI) M27-A3 and EUCAST EDef 7.3 microdilution broth procedures (Clinical, Institute LS, 2008; Arendrup et al., 2012). The MIC_50_ and MIC_90_ values (defined as the concentration that inhibit 50% and 90% of isolates tested, respectively) of ibrexafungerp compared to untreated control were 0.125 and 0.25 μg/ml, and 0.5 and 1 μg/ml for CLSI and EUCAST, respectively. In contrast, the MIC_50_ and MIC_90_ of fluconazole were 2 and 4 μg/ml, and 16 and 32 μg/ml CLSI and EUCAST, respectively, while the MIC_50_ and MIC_90_ of micafungin were ≤0.007 and ≤0.015 μg/ml, and 0.125 and 1 μg/ml CLSI and EUCAST, respectively (**Table 3**).

**Table 3:** MIC values (μg/ml) of ibrexafungerp and comparators against 31 C*. glabrata* isolates using two microdilution broth procedures, the clinical and Laboratory Standards Institute (CLSI) M27-A3 and EUCAST EDef 7.3 (Pfaller M., et al., 2012).

	CLSI M27-A3			EUCAST EDef 7.3		
	Ibrexafungerp	MICA	FLZ	Ibrexafungerp	MICA	FLZ
MIC_50_	0.125	≤0.007	2	0.25	≤0.015	4
MIC_90_	0.5	0.125	16	1	1	32

In the same study, sessile MICs were assessed by 50% and 80% reduction in the metabolic activity of the biofilm following antifungal treatment (SMIC_50_ and SMIC_80_) compared to growth control. The SMIC_50_ and SMIC_80_ values of ibrexafungerp in its ability to inhibit the growth of 50% of the 31 C*. glabrata* isolates tested were both 0.25 μg/ml, while the SMIC_50_ and SMIC_80_ required to inhibit growth in 90% of the isolates were 0.25 and 16 μg/ml, respectively. Ibrexafungerp showed higher activity compared to fluconazole, which had a SMIC_50_ and SMIC_80_ value of ≥256 μg/ml in its ability to inhibit the growth of 50% and 90% of *C. glabrata* isolates. Micafungin showed similar activity to ibrexafungerp, with SMIC_50_ and SMIC_80_ values of ≤0.015 μg/ml when inhibiting 50% of *C. glabrata* isolates, and SMIC_50_ and SMIC_80_ values of 0.25 and 2 μg/ml, respectively, when inhibiting 90% of isolates (**Table 4**) (Marcos-Zambrano et al., 2017).

**Table 4:** Sessile MICs at 50% and 80% reduction in the metabolic activity of the biofilm of 31 C*. glabrata* isolates (SMIC50 and SMIC80) following antifungal treatment compared to growth control (Pfaller M. et al., 2012).

	SMIC_50_			SMIC_80_		
	Ibrexafungerp	MICA	FLZ	Ibrexafungerp	MICA	FLZ
50% of Isolates	0.25	≤0.015	≥256	0.25	≤0.015	≥256
90% of Isolates	0.25	0.25	≥256	16	2	≥256

### Activity of Ibrexafungerp *In Vivo*

Ibrexafungerp has shown efficacy in *in vivo* models (Lepak et al., 2015; Wiederhold et al., 2018). Wiederhold et al. assessed the efficacy of ibrexafungerp administered orally against both wild-type and echinocandin-resistant *C. glabrata* infections in a neutropenic mouse model (**Table 5**). Mice were treated with either ibrexafungerp (including 30 or 40 mg/kg), caspofungin (1 mg/kg), or placebo for seven days. Ibrexafungerp at 30 mg/kg significantly reduced the kidneys fungal burden [number of colony forming units (CFUs)/g] in mice infected with both wild-type and echinocandin-resistant isolates compared to placebo (*P*<0.01). The 40 mg/kg dose of ibrexafungerp also significantly reduced the kidney fungal burden compared to placebo but only against the echinocandin-resistant isolate (*P*<0.01). The plasma concentrations of ibrexafungerp were also above the MICs for both wild-type (0.25 μg/ml) and echinocandin-resistant (1 μg/ml) isolates treated with the 30 and 40 mg/kg doses (combined mean 3.225 ± 1.023 and 3.683 ± 0.761 μg/ml, respectively) (Wiederhold et al., 2018). In contrast, Ghannoum et al., have shown that mice infected with *C. glabrata* and treated with ibrexafungerp doses ≤ 30 mg/kg showed significant reductions in kidney fungal tissue burden in both echinocandin-susceptible and -resistant strains of *C. glabrata* (Ghannoum et al., 2019).

**Table 5:** Assessment of the efficacy of ibrexafungerp and a comparator against both wild-type and echinocandin-resistant *C. glabrata* infections in a neutropenic mouse model (Lockhart et al., 2012).

Group	Dose	Mean log_10_ CFU/g ± SD			
		Wild-type	*P*-value vs. Placebo	Echinocandin-resistant	*P*-value vs. Placebo
**Placebo** (Methylcellulose)		5.58 + 1.11	——–	3.99 + 1.04	——–
**Ibrexafungerp**	30 mg/kg	3.57 ± 0.79	<0.01	2.38 ± 0.66	<0.01
	40 mg/kg	4.18 ± 0.96	*P*=0.074	2.34 ± 0.60	<0.01
**Caspofungin**	1 mg/kg	2.74 ± 0.76	*P*<0.01	3.61 ± 1.22	>0.05

Another study assessed ibrexafungerp activity against a neutropenic murine model of invasive candidiasis caused by *C. glabrata*. Four *C. glabrata* isolates were tested against four oral doses, 3.125, 12.5, 50, and 200 mg/kg of ibrexafungerp salt. Ibrexafungerp demonstrated potent activity *in vivo* against each isolate with a stasis endpoint achieved at a mean dose of 58.4 mg/kg. MIC range varied 8-fold (range, 0.03 to 0.25 μg/ml) achieving a mean tAUC/MIC of 315, and an fAUC/MIC of 0.63. The drug showed relatively linear pharmacokinetics over the dose range with a maximum concentration of drug in serum (*C_max_*) increased from 0.04 μg/ml to 2.66 μg/ml over the dose range. A concentration-dependent killing was demonstrated without paradoxical effect over the dose range. Protein binding was 99.8% and the elimination half-life ranged from 5.9 h to 8.5 h in plasma (Lepak et al., 2015).

## Clinical Efficacy of Ibrexafungerp

Another phase 3 open-label, single-arm study showed that oral ibrexafungerp provides favorable therapeutic response when used to treat refractory or intolerant *Candida* infections, namely *C. glabrata*. Of the 17 patients with invasive candidiasis/candidemia caused by *C. glabrata*, nine showed complete or partial response (52.9%), five had stable disease (29.4%), and three had their disease progress further (17.7%). Ibrexafungerp was also well-tolerated in patients with the most common adverse effect being gastrointestinal (Alexander et al., 2013).

## Summary and Conclusions

Over the past two decades, *C. glabrata* has become one of the most common species within the *Candida* family causing fungal infections. *C. glabrata* has become a growing concern for mucosal and systemic bloodstream infections and is particularly worrisome because of increasing multidrug resistance to both azoles and echinocandins. Novel antifungal agents in development, such as ibrexafungerp, a first-in-class triterpenoid antifungal, will provide treatment options to address these concerns. Ibrexafungerp allows the advantage of oral administration, unlike prior echinocandins, and is generally safe and well-tolerated with gastrointestinal adverse effects most commonly reported. Several studies have shown equal or superior *in vitro* activity compared to echinocandin antifungal drugs, such as caspofungin and micafungin, against both wild-type and echinocandin-resistant *C. glabrata* isolates. The *in vitro* activity of ibrexafungerp has also shown to be efficacious in low pH environments, which suggests its effectiveness in treating vulvovaginitis. Additional preclinical and clinical studies will be required to further confirm the efficacy and safety of ibrexafungerp in treating fungal infections caused by *C. glabrata* and other causes of invasive fungal diseases.

## Author Contributions

All authors listed have made a substantial, direct, and intellectual contribution to the work and approved it for publication.

## Funding

This work was supported by a grant from the National Institutes of Health (NIH grant # R21 AI143305-01). 

## Conflict of Interest

In compliance with the ICMJE uniform disclosure form, all authors declare the following: financial relationships: MG declares that he received funding from Pfizer, Scynexis, Inc., Cidara Therapeutics, and Amplyx Pharmaceuticals. Authors KBE and DA were employed by Scynexis, Inc.

The remaining authors declare that the research was conducted in the absence of any commercial or financial relationships that could be construed as a potential conflict of interest.
